# If fear of infertility restricts contraception use, what do we know about this fear? An examination in rural Ethiopia

**DOI:** 10.1186/s12978-021-01267-9

**Published:** 2022-06-13

**Authors:** Erica Sedlander, Hagere Yilma, Dessalew Emaway, Rajiv N. Rimal

**Affiliations:** 1 Department of Health Science, Boston University Sargent College, Boston, MA USA; 2 John Snow, Inc./ Last ten kilometers (L10K) Project, Addis Ababa, Ethiopia; 3 Johns Hopkins University Bloomberg School of Public Health, Baltimore, USA; 4 Department of Family and Community Medicine, University of California San Francisco, San Francisco, USA

## Abstract

**Background:**

Ethiopia has made great progress toward reducing unmet need for family planning and increasing contraception use over the last decade. However, almost one-quarter of women still have an unmet need. The primary reason for non-use is “method-related health concerns” and, within this broad category, the belief that using contraception will cause infertility is common. This belief extends beyond Ethiopia to low-, middle-, and high-income countries across the world. The objective of this paper is to examine associations with the belief that contraception use causes infertility and to subsequently suggest potential strategies to address this misperception.

**Methods:**

We collected data from women of reproductive age (between 15 and 49 years old) in 115 rural districts of Ethiopia (n = 706). Our main outcome variable was the belief that contraception causes infertility. We analyzed data, both individual-level factors and interpersonal factors, using nested logistic regression models.

**Results:**

Almost half of women in our sample (48.2%) believed that contraceptive use causes infertility. In the final model that included factors from both levels, self-efficacy to use contraception (AOR = 0.81, *p* < 0.05), visiting a health center and speaking to a provider about family planning in the last 12 months (AOR = 0.78, *p* < 0.05), and husband support of contraception (AOR = 0.77, *p* < 0.01) were associated with a reduction in the odds of believing that contraception causes infertility. The belief that infertility will result in abandonment from one’s husband (AOR = 3.06, *p* < 0.001) was associated with an increase in the odds of holding the belief that contraception causes infertility. A home visit in the last 12 months from a health worker who discussed family planning was not associated with the belief that contraceptive use causes infertility.

**Conclusions:**

Given that this belief is both salient and positively associated with other fears such as abandonment from one’s husband, it is critical for family planning programs to address it. Communication campaigns or interventions that address this misperception among couples may diminish this belief, thereby increasing contraception use and reducing unmet need in rural Ethiopia.

## Plain language summary

Qualitative research in sub-Saharan Africa has shown that women’s belief that contraception use causes infertility is a barrier to contraception use. In this paper, we examine different factors related to this belief and suggest strategies to address this misperception. We surveyed 706 married women from 115 rural districts of Ethiopia. We found that women who believed that infertility would result in abandonment from one’s husband had three times higher odds of believing that contraception causes infertility. We also found that some factors associated with a decreased odds in holding this belief included self-efficacy to use contraception, visiting a health center and speaking to a provider about family planning, and husband support of family contraception. A home visit from a health extension worker who discussed family planning was *not* associated with holding this belief. Our findings suggest some ways to address this misconception. Clearly, women’s own self-efficacy, or believing that they can use family planning, is an important piece to any intervention. Given that husbands’ support of contraception is associated with reduced odds of holding the belief that contraception causes infertility, including them in intervention efforts is also a logical step. Finally, a home visit from a health extension worker was not associated with reduced odds of holding this belief. Including information that contraception does not cause infertility and discussing the real causes of infertility as part of their education strategy may help debunk this myth and thereby reduce unmet need in rural Ethiopia.

## Background

The 2019 Ethiopian Demographic and Health Survey showed that, in the prior 15 years, married women in Ethiopia almost tripled their use of modern contraception [1]. Increased adoption of modern contraceptives can delay the onset of childbearing, space births, and limit completed family size. In turn, a resulting decline in fertility may lead to economic improvements, better health outcomes for women and children, and improved gender equality [2]. Therefore, reducing unmet need for modern contraceptives remains a top priority for organizations working in global health and international development [3, 4]. Some of the factors attributed to Ethiopia’s rapid increase in family planning use include growing political will, substantial external funding, nongovernmental and public–private partnerships, and the implementation of a large health extension worker program [5, 6].

Despite this progress, 22% of married women in Ethiopia still have an unmet need for family planning [7]. Although knowledge of contraceptive methods is almost universal and access barriers are declining, demand-side barriers persist [8]. The most recent full demographic health survey shows that 18% of Ethiopian women reported that they stopped using contraception due to “method-related health concerns.” However, the measure does not ask about *which* specific concerns women have [7].

Common contraception-related health concerns include the belief that contraception may cause cancer, change menstrual bleeding, promote weight gain, and result in infertility [9–11]. The belief that using contraception causes infertility has been reported both qualitatively and quantitatively as one of the primary reasons for not using long-acting contraception in Northern Ethiopia [12]. Gebremariam and Addissie [12] found that more than one-quarter (26.2%) of participants perceived that contraceptive methods could “harm the womb.”

This belief is not unique to Ethiopia. A 2020 scoping review of fear of infertility in Africa found 15 qualitative studies that cited the belief that contraception causes infertility [13]. Specific studies cited the belief that contraception can cause structural damage to a woman’s reproductive organs. This belief also reaches beyond Africa. Studies in the United States [14, 15], Guatemala [16], Turkey [9], Bangladesh [17, 18], and Vietnam [19] reported that this fear is a barrier to contraception use. A systematic review of barriers to contraception use among young people in low to middle income countries reported that the belief that contraception use would cause infertility was the most cited reason for non-use [20]. And in our recent research in rural Kenya, we found that holding this belief was associated with reduced odds of using contraception. Furthermore, if a man or woman's social network holds this belief, there is an even greater reduction in the odds of using contraception [21]. Although the belief around contraception and infertility is salient and pervasive, it is surprising that, to our knowledge, no peer-reviewed studies have examined factors associated with this fear itself. Our study provides initial ideas. The objective of this paper is to identify multilevel factors associated with the belief that contraception use causes infertility and to inform approaches to address this barrier.

### Conceptual model

**Fig. 1:**
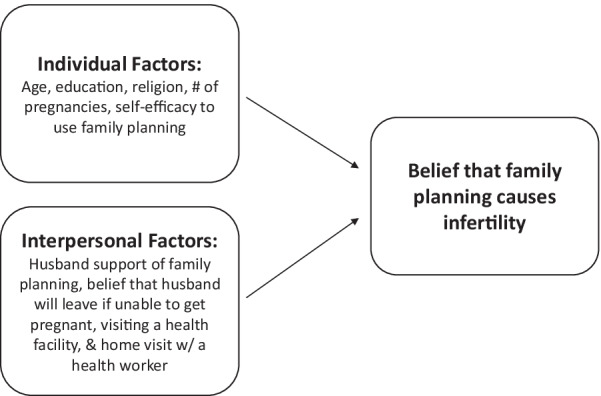
Conceptual model of the multi-level factors that are associated with the belief that contraception use causes infertility

To address the multilevel factors that may affect this belief among women who are not using contraception, we use factors at two levels of the socio-ecological model to frame our work [22]. Individual-level beliefs about infertility that influence contraceptive use may diffuse within communities through interpersonal communication [23]. Prior research shows that contraceptive use is associated with factors at multiple levels, including those at the individual level (e.g., education and attitudes); interpersonal level (e.g., husband’s support for family planning); structural level (e.g., interaction with the frontline health worker system); and socio-normative level (e.g., collective norms, which is the prevalence of an attitude, belief, or behavior within a group) [24–28]. We chose to use nested models to examine which variables are associated with this belief at two different levels of the socio-ecological continuum. Examining these different levels separately and then altogether allows us to document how the associations change as we introduce higher level variables into the model (see Fig. 1).

## Methods

### Study setting

Ethiopia is in the horn of Africa, composed of nine regions with about 95 million people. This study was conducted in 115 woredas (districts) in the four most populous regions of Ethiopia: Amhara; Oromia; Southern Nations, Nationalities, and People’s (SNNP); and Tigray, where John Snow Inc Research & Training Institute (JSI) was implementing the Last Ten Kilometers Project funded by the Bill & Melinda Gates Foundation. The intervention area covered about 19% of the population in Ethiopia [29]. The Last Ten Kilometers (L10K) aimed to contribute towards the achievement of the post Millennium Development Goals related to maternal and child health in Ethiopia through enhanced interactions among households, communities, and the Health Extension Program [29].

The primary health care system of rural Ethiopia comprises a district hospital and three to four health centers, each with five satellite health posts. The health posts have two female health extension workers (HEWs), who are a part of Ethiopia’s flagship Health Extension Program, each serving a community (kebele) of about 5000 people with basic community-based health services including family planning.

### Study design and participants

We conducted a cross-sectional survey of women of reproductive age (15–49 years) in early 2016 representing the L10K intervention area. Study participants were married women not using any family planning method during the survey. We obtained ethical clearance from Amhara, Oromia, SNNP, and Tigray Regional Health Bureaus and from JSI.

### Sampling

The sample size for women of reproductive age was based on L10K program evaluation needs. We implemented a two-stage cluster sampling design to obtain the required sample. At the first stage, we randomly selected 301 kebeles/communities with the probability proportional to their population sizes. At the second stage, we selected households. To do so, we subdivided a kebele into three equal segments; from each segment, the quota was to interview four women of reproductive age. We randomly selected the first household from each segment. Every fifth household was visited and all women of reproductive age in the visited household were interviewed until the quota for each segment was fulfilled.

The study team translated a structured questionnaire into the three major local languages (Amharic, Oromifa, and Tigrigna). Survey data were collected by the field teams using smart phones. Verbal consents from respondents were sought and documented by interviewers prior to interviewing. If a respondent was younger than 18 years old, consent was sought from her husband or guardian. Because many respondents were not able to read or write, written consents were not obtained. If the respondent agreed to be interviewed upon listening to the consent statement, the interviewer electronically marked the questionnaire as consent given and only then continued with the interview.

### Inclusion criteria

For this paper, we analyzed data from women (n = 706) who answered a question about the belief that contraception may affect their future fertility. This question was only asked of women who were not using family planning, which represents approximately 19% of the total sample. See the L10K final report for a more detailed account of data collection methods [30].

### Measurement

Our individual level variables included age, education, number of prior pregnancies, religion, and self-efficacy to use family planning. Self-efficacy was measured as the response to one question that asked for level of agreement on a four-point Likert scale: “I am confident I can use family planning methods and avoid pregnancy until I want to get pregnant.”

We also included interpersonal variables such as husband support of family planning (measured on dichotomous scale “yes” or “no”), and belief that the respondent’s husband will leave her if she were infertile (measured on a four-point Likert scale from “strongly agree” to “strongly disagree”). Responses to these questions were recoded so that higher values indicated stronger perceptions of husband support and stronger belief that infertility would cause husbands to leave. The question about whether a woman’s husband would leave if she was infertile was only asked of women who were not using family planning. Additionally, we included any interaction with a health worker in the last 12 months as an interpersonal-level variable. We measured interaction with a health worker with the following question: “In the last 12 months, were you visited by a community health worker who talked to you about family planning?” coded with a dichotomous “yes” or “no” response. Finally, we included a variable about visiting a health facility in the last 12 months when the provider spoke about family planning: “If yes (you visited a health facility for yourself in the last 12 months), did any staff speak to you about family planning methods?” was coded with a dichotomous “yes” or “no” response.

We assessed our outcome variable, the belief that using family planning will affect fertility, with one question measured on a four-point scale from “strongly agree” to “strongly disagree”: “If I begin using a family planning method, I'm afraid I won't be able to get pregnant after that—even when I want to.” Responses were dichotomized such that those who indicated that they “agree” or “strongly agree” with this belief were given a score of 1, and those that indicated they “disagree” or “strongly disagree” were given a score of 0. This question was only asked among women who were not using family planning. Given that this is our main outcome variable and some of our independent variables were also only asked among non-users, we only included women who were not using family planning in our sample.

### Statistical analysis

We conducted our analyses in four steps. First, we calculated descriptive statistics. We then performed bivariate Pearson’s Zero-Order correlations and multivariable logistic regressions analyses to identify factors associated with the belief that contraception use causes infertility. We show nested models to demonstrate how each level is differentially associated with this belief. The first model contained individual-level factors, after which we added interpersonal factors. All variables were standardized before being entered into the nested models. We used STATA version 14 to conduct all analyses.

## Results

Description of the sample included in our study is shown in Table 1. Participants’ average age was 30 years, (59.6%) could not read/had no schooling, 29% completed primary school, and (14.3%) continued after primary school. Less than (1%)of the sample had no prior pregnancies, (24.9%) had one or two pregnancies, 34.6% had three or four, and (39.8%) had five or more. Only (5%) of the sample was not married (n = 207), so we did not include unmarried women in the model. Almost the entire sample, (92.3%), reported that they felt confident they could use family planning methods and avoid pregnancy until they wanted to get pregnant. Almost one-quarter of the women, (24.6%), had been visited by a health worker in the last 12 months who talked to them about family planning. Almost one-third, 31.3%, had visited a health center in the last 12 months where the provider spoke to them about family planning. Almost (40%) reported that if they were unable to get pregnant, they would be afraid that their husband would leave them. Most women, (84.1%), reported that their husbands supported family planning. Almost half (48.2%) of women reported that they believe that using family planning will affect fertility.

**Table 1:** Description of the sample (married women ages 15–49 years) in Ethiopia (n = 706)

	*M (SD)*
Age	29.7 (6.44)

	*n (%)*
School	
None	421 (59.6)
Completed primary	184 (29.0)
Higher than primary	101 (14.3)
Religion	
Orthodox	488 (69.1)
Muslim	136 (19.2)
Protestant	81 (11.4)
Number of pregnancies	
Zero	5 (0.71)
One or two	176 (24.9)
Three or four	244 (34.6)
Five or more	281 (39.8)
Has self-efficacy to use family planning methods	667 (92.3)
Visited by health worker in the last 12 months who spoke to them about family planning	174 (24.6)
Visited a health facility in the last 12 months and spoke about family planning	221 (31.3)
Husband supports contraceptive use	589 (84.1)
If infertile, afraid husband will leave them	282 (39.9)
Believes contraceptive use causes infertility	340 (48.2)

Table 2 shows the zero-order correlations that indicate that fear of infertility was significantly associated with self-efficacy to use family planning (r = − 0.14,* p* < 0.001), husband support of family planning (r = − 0.17,* p* < 0.001), and fear that husband will leave if one is infertile (r = 0.47,* p* < 0.001).

**Table 2:** Zero-order Pearson correlations

	1	2	3	4	5	6	7	8	9	10
1 Belief CU causes infertility	1.00									
2 Age	− 0.03	1.00								
3 Education	− 0.01	− 0.30***	1.00							
4 Religion	0.05	0.01	− 0.04	1.00						
5. Number of pregnancies	0.00	0.75***	− 0.35***	0.12**	1.00					
6 Self efficacy	− 0.14***	− 0.07	0.16***	0.02	− 0.13***	1.00				
7 Health worker home visit	− 0.04	− 0.05	0.02*	− 0.05	− 0.02	0.07	1.00			
8 Health facility visit	− 0.04	− 0.09*	0.10**	− 0.02	− 0.07	0.05	0.37***	1.00		
9 Husband supports CU	− 0.17***	− 0.07	0.05	− 0.05	− 0.03	0.16***	0.11**	0.12**	1.00	
10 Husband leave if infertile	0.47***	− 0.03	− 0.04	− 0.03	− 0.00	− 0.13***	− 0.00	0.13***	− 0.12*	1.00

*CU* contraceptive use

*p < 0.05, **p < 0.01, ***p < 0.001

Logistic regressions (Table 3) showed that in the *individual*-level model, only self-efficacy to use family planning (AOR = 0.74,* p* < 0.001, 95% CI [0.63–0.87]) was associated with a reduced odds in believing that contraception affects fertility. The more confidence women reported in using contraception, the less likely they were to hold the belief that it causes infertility.

**Table 3:** Multivariable associations with the belief that contraceptive use will cause infertility in married Ethiopian women who are not using contraception, from logistic regression equations

Ethiopian Women ages 15–49 years	*(N* = 700)			
	Individual	95% CI	Interpersonal	95% CI
Age	0.98	[0.94–1.01]	0.97	[0.93–1.01]
Education	1.00	[0.85–1.18]	1.08	[0.89–1.30]
Religion	1.11	[0.96–1.30]	1.19	[1.00–1.43]
Number of pregnancies	1.03	[0.93–1.14]	1.04	[0.92–1.18]
Self-efficacy contraceptive use	0.74***	[0.63–0.87]	0.81*	[0.68–0.98]
Health worker home visit in last 12 months and spoke about CU	1.01			[0.84–1.22]
Health facility visit in the last 12 months and spoke about CU			0.78*	[0.65–0.95]
Husband supports contraceptive use			0.77**	[0.65–0.93]
Husband will leave if infertile			3.06***	[2.52–3.72]
(Pseudo r-squared)	(0.02**)		(0.20***)	

Odds ratios are from logistic regression equations, when all main-effects have been entered. *CU* contraceptive use. *p < 0.05, **p < 0.01, ***p < 0.001

We then added four *interpersonal*-level variables: receiving a visit from a health worker who spoke about family planning in the last 12 months, visiting a health center where the provider spoke about family planning, husband support of family planning, and fear that husband will leave for reasons of infertility. This model showed that self-efficacy was still associated with reduced odds of holding the belief that contraception causes infertility (adjusted odds ratio (AOR) = 0.81,* p* < 0.05, 95% confidence interval (CI) [0.68–0.98]). Also, husband support of family planning (AOR = 0.77,* p* < 0.01, 95% CI [0.65–0.93]) and visiting a health worker who spoke about family planning (AOR = 0.78,* p* < 0.05, 95% CI [0.65–0.95]) were also associated with reduced odds of holding this belief. On the other hand, the fear that your husband will leave if you are infertile was associated with an increased odds in this belief (AOR = 3.06,* p* < 0.001, 95% CI [2.52–3.72]).

## Discussion

In this study, we found that self-efficacy to use family planning, husband support of family planning, visiting a health center in the last 12 months, and belief that a husband will leave if unable to get pregnant are significantly associated with the odds of holding the belief that modern contraception impacts fertility. We also found that a visit from a health worker in the last 12 months who discussed family planning was *not* significantly associated with the belief. To our knowledge, this is the first study to quantitatively examine factors associated with this belief.

Although the belief that contraception use causes infertility has been documented in many parts of the world, to our knowledge, past communication programs in sub-Saharan Africa have rarely directly addressed it [31–33], despite calls to do so [11, 13, 34, 35]. Hence, we know relatively little about what types of interventions are able to allay the fear that contraception use can make one infertile.

Even though fear of infertility is an individual-level phenomenon, it is interesting that individual-level variables in our model yielded a small pseudo r-squared. Only when interpersonal factors were added to the model did the pseudo r-squared increase. Although interpretation of the pseudo r-squared must be made with caution, this finding may indicate that this individual-level fear is grounded in higher-level factors. In our recent work in rural Kenya, we similarly found that higher level factors had a greater effect than individual factors. Specifically, we found that one's social network beliefs that contraception use causes infertility affected individual contraception use even more than one's own beliefs. Within this study, at the interpersonal level, fear about husbands’ reactions was significantly associated with fear of infertility. This association paints a rather grim picture: women who were afraid of becoming infertile had two reasons to be fearful—that their husbands would leave them and that taking modern contraceptives would further exacerbate the situation. Looking at it from another perspective, our findings seem to suggest that, for many women, use of modern contraceptives was associated with two negative outcomes—that one would become infertile and that, as a result, one’s husband would leave. Other studies in sub-Saharan Africa have also reported that women fear that their husband will leave them if they are infertile [11, 36]. Tilson and Larsen [37] examined national Ethiopian data and found that having a child within the first marriage was significantly associated with a reduced risk of divorce [37]. Clearly, this fear is not limited to our study and not unfounded.

We also found that a home visit from a health worker who discussed family planning was *not* associated with the belief that modern contraception results in infertility. This finding suggests that debunking this misperception was perhaps not a part of the HEW curriculum. Including this in their annual training, continued education, and training curriculum may change misperceptions. Furthermore, as health workers often come from the communities they serve, some health workers may believe it themselves. Educating them about the seriousness and prevalence of this misinformation may be a logical step for future interventions. However, it is important to note that this variable was measured as a dichotomous indicator, in which any interaction with a health worker in the past 12 months was recorded. It may be the case that the frequency of interactions with a health worker (and not just *whether* one had an interaction) can influence the belief that family planning can cause infertility. Unfortunately, we did not measure frequency of interaction.

However, we did find that visiting a health center where the provider spoke about family planning was associated with a reduced odds of holding the belief that modern contraception results in infertility. This finding implies differences in the type of information that women may be receiving from a health center visit as opposed to an HEW home visit. Indeed, HEWs’ curriculum, education, training, and so on varies from that of a health facility provider. HEWs may require more training and adjustments to their curriculum to be able to address this misperception.

The belief that infertility will result in abandonment from one’s husband was associated with an increased odds of holding the belief that modern contraception results in infertility. Demand-side family planning efforts thus need creative ways to include men in the conversation. Studies show that gender inequities and gender roles significantly affect contraception use [38]. Additionally, informing couples that infertility can be a result of both male and female factors may alleviate the burden on women [39]. Future interventions may also consider working with newly married couples to enable open discussion about family planning within the home and to improve individual self-efficacy to use family planning methods. Furthermore, a communication campaign for couples could acknowledge that using contraceptives and becoming a mother are not mutually exclusive, perhaps by role modeling mothers (or couples) who have previously used family planning methods.

Of course, we have a real concern that addressing this belief could exacerbate it even more. This fear may be one reason that interventions have largely ignored it despite a plethora of research reporting this barrier. To ensure that interventions are tailored to the community and effective, qualitative research, including human-centered design and monitoring with real-time intervention tweaks, may be an effective approach. A 2015 review provides several useful recommendations. For example, it may not be enough to simply discredit a myth without also replacing the myth with an alternative explanation [40, 41]. Thus, rather than merely stating that modern contraceptives do not cause infertility, interventions may wish to include information about the real causes of infertility. Bedsider.org, a United States–based organization, does just that in its communications [42].

In addition to intervening to address this misperception, we need to better understand the belief itself. Future research should examine the best way to measure the belief that modern contraception results in infertility. In our sample, almost half the women held this belief. However, we do not know if these beliefs refer to permanent infertility or delayed infertility. If the beliefs are around delayed infertility, how long after stopping contraception do women believe fertility will return? Prior studies have measured infertility in the following ways:

- “Contraceptives can harm a woman’s womb.” [12]
- “Combined oral contraception pills cause infertility.” [9]
- “Using medical methods of family planning can cause women to become infertile.” [43]
- “If I begin using a family planning method, I’m afraid I won’t be able to get pregnant after that—even when I want to.” [30]

Given the disparate measures, it is critical to improve how we measure this belief and then to use a consistent measure to compare across contexts and populations. To our knowledge, we know of no fertility measure that includes a time stamp. Another important aspect to understand is how this belief differs by method of contraception. Prior qualitative studies have shown that some methods are more linked to this belief than others [11, 13, 34]. Furthermore, prior studies only include women. It is important to understand how pervasive this belief is among men and between generations (e.g., mothers and mothers-in-law).

We must also acknowledge that real infertility exists. Infertility estimates in Sub-Saharan Africa range from 2 to 31% depending on location, population, and measurement method [44, 45]. This may explain the prevalence of the belief that contraceptive use can lead to infertility. When a couple has difficulty conceiving or is married for several years before conceiving, the entire community may notice. On the other hand, infertility may be confused with simply taking the normal time to conceive or even a couple’s desire to wait to conceive. Regardless, infertility needs to be addressed. In rural areas in sub-Saharan Africa, infertility can have devastating consequences for a woman. Research in sub-Saharan Africa [36, 46, 47] suggests that infertility remains a source of economic and social devastation for those who experience it, with such women being at risk of their husband leaving them or taking a second wife and prevented from attaining the status of full womanhood. Therefore, expecting women to use contraception—when they believe that it could result in infertility and potentially a host of negative ramifications—without addressing their misperception brings up ethical questions for interventionists working to simply increase contraception use. A more person-centered approach to family planning that considers each women’s lived experiences is necessary to truly reduce unmet need.

### Limitations

One limitation of this study is that the question about the belief that contraception use causes infertility was only asked of women who were not using contraception. It is likely that this belief also exists among women who are using contraception, who perhaps believe the benefits of preventing unintended pregnancy outweigh the potential costs of affecting fertility in the future. Additionally, because our sample only includes married women, almost all of them have at least one child. Women without any children likely hold this fear as well because they have not yet conceived and, thus, have not been able to demonstrate that they are fertile. Past research in rural Ethiopia has shown extreme pressure on newly married couples to conceive soon after marriage, sometimes in part to prove their fertility [48].

One of the reasons that individual factors may have accounted for so little variance is that we did not have measures of personal infertility/subfecundity or family infertility. Future studies of this belief should include personal experience with infertility and family experience with infertility. However, within our dataset, one of the responses to “reason for not using contraception” was subfecund/infecund, and only 10 of 706 women, or less than 2% of the sample, reported that they were subfecund/infecund.

Another limitation is that our measure of the belief that contraception affects fertility does not include any mention of the time it may take to get pregnant. There may be differences in women believing that contraception delays conception versus making it impossible altogether. Future research should examine these nuances because they are two separate beliefs: contraception delays fertility versus causes one to be infertile altogether.

Additionally, our measure does not include beliefs about different contraceptive methods. Beliefs may differ between methods (e.g., intrauterine devices versus injectables). Beyond not using contraception altogether or discontinuation, the fear that contraception causes infertility may affect switching methods to a method that is deemed to have less of an effect on future fertility. Future research should examine how beliefs differ among methods and time frames. Additionally, the study sampling involved clustering, but because we did not include any variables at the village or cluster level, we did not account for clustering in our analysis. The intraclass correlation (ICC) in our final sample for the outcome investigated here (fear of infertility) was 0.096, corresponding to a design effect of 1.60. Although there is debate in the literature about the magnitude of ICC effects, an ICC below 0.10 is generally considered to have small-to-medium effects [49]. Further, our design effect is below 2, indicating that the analysis includes enough women from each woreda to minimize biases due to clustering effects [50]. Finally, the cross-sectional nature of the study limits our conclusions to associations and not causal linkages. Future studies may want to design an intervention that addresses women’s concerns around contraceptive use including this belief and then evaluate which factors affect this belief over time and which strategies are most effective to address this misperception.

## Conclusions

In closing, policy and behavior-change program designers must understand and mitigate the impact that the fear of infertility has on a woman’s desire to use contraception. By successfully reducing the prevalence of the belief that contraceptives cause infertility, interventions could reach the last 22% of women in Ethiopia who still have an unmet need for contraception.

## Data Availability

The datasets generated and/or analyzed during the current study are not publicly available but are available from the corresponding author. You can also find all tools and reports from the study here: http://l10k.jsi.com/index.htm.
